# Frontiers of cancer care in Asia-Pacific Region

**DOI:** 10.2349/biij.4.3.e45

**Published:** 2008-07-01

**Authors:** CB Saw, JE Shogan

**Affiliations:** 1 Penn State Hershey Cancer Institute, Hershey, Pennsylvania, United States; 2 UPMC Cancer Centers, Pittsburgh, Pennsylvania, United States

The incidence of cancer has increased significantly over the past several years in the Asia-Pacific region. Cancer is one of the leading causes of death in South Korea, Taiwan, Singapore, Hong Kong as well as in urban China, and has become a major concern for many healthcare systems in the Asia-Pacific region [1]. This increase in cancer deaths has been attributed to the enormous economic successes in the East and Southeast Asia countries and China during the 1980s and 1990s, with impressive double-digit growth rates per year. Such economic successes have led to improved standards of living and healthcare systems resulting in fewer deaths from infectious diseases. For example, in the Seventh National Health Development Plan (1992-1996), the Thailand government had increased the health care budget by 54% in five years [1]. The increased wealth in the region has also allowed the private sectors to invest in healthcare services as demonstrated in Indonesia, and in Singapore with the restructuring and privatisation of its former public hospitals. The people in Asia are therefore living longer, expanding its population in a much more rapid rate compared to the population in Europe and the USA. In accordance with the statistics from the World Health Organization (WHO), the number of people worldwide who died from cancer in 2005 was 7.6 million out of 58 million overall. More than 70% of cancer deaths have been in the low and middle income countries, attributed to the limited availability of healthcare resources to prevent, diagnose, and treat cancer. The projected number of cancer deaths will increase to 9 million in 2015 and 11.4 million in 2030 [2].

Aside from the rapid increase of the general population in the Asia-Pacific region compared to Europe and USA, the proportion of people who are 65 years and older has been increasing at an even more alarming rate. For example, the population of China has increased by 31% over the last 25 years, while the number of people over 65 years has increased by 81%. By comparison, the population in UK has only risen by 6% and the number of people older then 65 years by 7% in accordance with the Lancet Asia Medical Forum highlights of the “Asia and Cancer Management in the 21^st^ Century” held in April 21-27, 2007 in Singapore [3]. These disparities are worrisome as cancers affect primarily people of older age. If the projection is correct, the number of cancer cases in Asia is set to increase from 3.5 million in 2002 to 8.1 million by 2020 if the current management strategies are not changed.

The management of cancers in the Asia-Pacific region poses many challenges. In addition to enjoying longer lives, the people in the Asia-Pacific region are also adopting bad lifestyle habits that greatly increase the risk of cancer. About twice as many men in Asia smoke as compared to the men in Europe and to the USA where steps are taken to limit or prohibit smoking. Selective infections, such as the Helicobacter Pylori, exist in 75% of the population in South-Central Asia, which increases the risk of stomach cancer by 5-6 times. Hepatitis B virus is endemic, substantially increasing the risk of liver cancer in Southeast Asia. Nasopharyngeal cancer, often identified as “Hong Kong Cancer”, is a very common disease in Asia and predominantly affects people of southern Chinese descent. Research has been ongoing to identify whether this disease is due to environmental factors, heredity, or otherwise. Exposure to sunlight also poses greater cancer risk. The global UV index for Asia is about twice that for the countries in the Northern Hemisphere. The economic growth has also led to rapid industrialisation and urbanisation, causing increased pollution which contributes to the increased risks in cancer, in particular for those types of cancers found in the West such as breast, lung, and prostate cancers. Lastly, a high-fat diet, drinking of alcohol, consumption of unhealthy food, and lack of physical exercise also increase cancer risk.

The rapid increase in cancer in the Asia-Pacific countries places a huge burden on the Asia-Pacific society as a whole. The healthcare systems set up by the government of each country has to include the management of cancers. Most developing countries have opened clinics and specialty hospitals for cancer therapy. The cancer management programs also include prevention, diagnosis and treatment, in addition to the mechanism of payment for services such as health insurance. The cultural expectations from the public have to be addressed as well. Far fewer patients undergoing treatment are generally cured and this failure is often reflected as the failure of the oncologists.

Cancer screening programs must be developed to detect early cancers to improve survival. Likewise there must be sufficient treatment facilities with appropriate experts and personnel to manage its cancer patients undergoing treatments in each country. While it has been presumed that different types of cancer predominate in different countries, the documentation of such patterns is still lacking. Likewise, the treatment preferences have been seen to vary from country to country or region to region. The presumed differences have been echoed by Dr. Franco Cavalli, President of the UICC who spoke at the Lancet Asia Medical Forum [3]. The president commented: “Speakers from outside Asia tend to overlook the huge differences which exist not only in terms of the causes of cancer, but also in terms of the management between the western countries and Asia. Conditions such as the structure of healthcare systems are very different from one country to the other. It is therefore necessary to adapt the discussion to local situations.” [4]

Practising oncologists in each country of the Asia-Pacific are invited to contribute an article (without restrictions - update, review or original work) on cancer care in their country to collectively compile a special issue in the Biomedical Imaging and Intervention Journal (biij). biij is an open access peer-reviewed online journal and the articles are available to the public, in particular healthcare providers, healthcare administrators, government officials, investors, patient advocates, and practising oncologists and associated staff. This special issue entitled “Frontiers of Cancer Care in Asia-Pacific Region” is intended to provide the oncologists in Asia-Pacific the unique opportunity to present and hence, share their strategies of handling cancer patients in their individual country, in particular the attempts by the government and the private sectors to improve cancer care services, the philosophy of cancer care as well as the need for specialised staffing. Besides these invited articles, this special issue also contains articles of special interests: (1) translational research concept, (2) innovations of therapies, (3) informatics, (4) quality assurance processes, and (5) multi-discipline support from the medical physicists and nurses. These topics are chosen as complementary contributions to the articles by the oncologists from the Asia-Pacific. For example, research and participation in clinical trials in the Asia-Pacific are still limited and will continue to increase [3]. The understanding with the basic knowledge of translational research concept and mechanisms certainly would be helpful in carrying out these research programs.

The dedication of the authors from the Asia-Pacific countries deserves recognition for the successful compilation of this special issue of biij. First, the clinical practice of the oncologists in the Asian countries is generally very busy. The writing of these articles involves the sacrifice of personal time. The guest editors would like to thank the authors for investing a significant amount of their time and effort in writing these articles as well as allowing the manuscripts to undergo review. The second obstacle imposed on the authors is the translation of information into English. This is particularly difficult if English is not routinely used in their clinical practice. The third concern is the exposure of in-depth knowledge of the authors’ clinical practice. Because of these struggles and intimidating concerns, some oncologists have declined to contribute. For those who are willing to participate and share their experiences, this signifies their strong support for our vision to provide a common platform of presentations aimed at sharing knowledge with the unified goal of effective cancer care in the Asia-Pacific region. It is the hope of the guest editors that the articles in this special issue will contribute to the understanding of clinical practice for oncologists within as well as between the neighboring countries in the Asia-Pacific region to promote effective disease management with limited resources and/or infrastructure. The fruition of this special issue signifies our first call or attempt to address cancer care in the Asia-Pacific as a region and to discern similarities and differences. It is the hope of the guest editors that future calls will increase the participation of more oncology colleagues in the Asia-Pacific. Again, the guest editors would like to thank the authors and co-authors for making their contributions and the honorary editor (Dr. Kwan-Hoong Ng) for consenting to this special issue as part of the biij publication.
